# Effectiveness versus Uptake: The Challenges of Implementing Evidence-Based Strategies to Reduce Surgical Site Infection in Patients with Colon Surgeries

**DOI:** 10.1089/sur.2022.411

**Published:** 2023-04-27

**Authors:** Lena Camperlengo, Maureen Spencer, Peter Graves, Walter Danker, Charles E. Edmiston

**Affiliations:** ^1^ Premier, Inc., Charlotte, North Carolina, USA.; ^2^ Infection Preventionist Consultants, Boston, Massachusetts, USA.; ^3^ Clinical Solution, LLC., Corinth, Texas, USA.; ^4^ Johnson & Johnson, Somerville, New Jersey, USA.; ^5^ Medical College of Wisconsin, Milwaukee, Wisconsin, USA.

**Keywords:** colorectal, guidelines, implementation, standardization, SSI, sutures

## Abstract

**Background:**

National and international recommendations for the prevention of surgical site infection (SSI) were published six years ago, but little is known about implementation in colon surgeries.

**Methods:**

We conducted an observational study to evaluate the implementation of seven SSI-prevention elements in colon surgeries. Study coordinators recorded the implementation using an electronic case report. Surgeons completed a survey that identified key drivers of implementation. Three peer-to-peer calls and a study coordinator survey provided insights on the obstacles and drivers to implementation.

**Results:**

The elements ranged in compliance from 100% to below 1%. Absence of documentation in the electronic medical record (EMR), conflicting local policies, and a lack of standardization of processes and products were significant obstacles in implementation.

**Discussion:**

Standardizing peri-operative procedures may be accomplished by implementing guidelines. Using implementation science to reduce variability and stocking leads to product standardization with items that support evidence-based practices. Administration, material management, and surgical leadership all have a duty to the patient to reduce obstacles to implement evidence-based practices.

**Conclusions:**

Our study reveals variability in in the integration of published guidelines into clinical practice. Every surgical patient deserves the best possible care by using evidence-based guidelines and practices centered on reducing SSIs.

Surgical site infection (SSI) after colorectal surgery is a common complication associated with poor outcomes, longer length of stays, and increased re-admissions.^1^ Reports indicate that up to 55% of infections in patients who had colorectal surgery could have been prevented.^2,3^ In 2016, the World Health Organization (WHO)^4^ and the American College of Surgeons (ACS)^5^ published evidence-based guidelines to reduce and prevent SSIs. The following year, the U.S. Centers for Disease Control and Prevention (CDC) published their SSI-prevention guidelines.^6^

The use of published guidelines,^5–9^ standardized SSI definitions, and surveillance reporting^10–13^ demonstrate benefit in colorectal SSI reduction. However, implementing them poses challenges, as does sustaining new behaviors.^14^ Key elements of traditional interventions to prevent SSIs include implementing or improving a safety culture, using data tracking and feedback mechanisms, and using checklists or evidence-based bundles.^15^ Despite focusing on reducing morbidity and mortality, hospitals in the National Healthcare Safety Network (NHSN) that perform colorectal surgeries demonstrated a modest decrease (5%) in 30-day SSIs in 2020 compared with 2019.^16^

The use of implementation science (IS) promotes timely behavioral changes among individuals and groups. Implementation science methods include identifying obstacles and drivers across multiple levels of the healthcare continuum and developing and applying strategies to increase adoption of evidence-based clinical recommendations.^17^ Using IS as our lens, we evaluated the implementation of seven SSI-reduction elements from three guideline sources published approximately six years before the study began (Table 1). We sought to understand better the obstacles and drivers to implementing these evidence-based practices in a real-world setting.

**Table 1.** Seven Studied Colon SSI Prevention Elements from Published Guidelines

Studied elements from guidelines	Abbreviation	Where published^a^
Administering a weight-dependent dose of pre-operative IV antimicrobial agents	IV antibiotic agents	WHO, ACS, CDC
Using triclosan-coated sutures at the deep layer, organ layer, and superficial layer	Triclosan sutures	WHO, ACS, CDC
Controlling a patient's blood glucose at or below 200 mg/dL peri-operatively	Blood glucose	WHO, ACS, CDC
Maintaining the patient's body temperature above 36.5°C once under care	Body temperature	ACS, CDC
Placing the patient on oxygen beginning in the pre-operative period until at least 2 h after waking in the post-operative period (delivered with nasal cannula at a minimum of 3 L/min)	Oxygenation	WHO, ACS, CDC
Application of a topical skin antiseptic: 2% CHG/70% isopropyl alcohol (ChloraPrep); or 4% aqueous CHG (generic); or aqueous povidone iodine (generic); or 74% isopropyl alcohol/iodine povacrylex (Duraprep) or Para-chloro-meta-xylenol (PCMX)	Skin preparation	WHO, ACS, CDC
Ordering mechanical bowel preparation and oral antibiotic agents before surgery	MBP + oral ATBs	WHO, ACS

IV = intravenous; CHG = chlorhexidine gluconate; MBP = mechanical bowel preparation; ATB = antibiotic agent.

^a^ World Health Organization (WHO); American College of Surgeons (ACS); Centers for Disease Control and Prevention (CDC)

## Methods

### Study design

We conducted an observational study using mixed methods to evaluate the implementation of seven SSI-prevention elements at three hospitals (Table 1). Each element was chosen and operationalized in the peri-operative setting to include pre-operative orders, and practices used in the operating room and post-anesthesia acute care unit. The study population represented approximately 800 colon surgeries over a 12-month period. Study sites were three large hospitals from geographically diverse locations in the United States. Site one was in the north-central United States in a large urban setting. Site two was in the southern United States in an urban setting. Site three was in a Midwest city. All three hospital sites were chosen for their volume of at least 250 colon surgeries per year.

A centralized Institutional Review Board (IRB) approved the study protocol and consenting process for clinician participants. Clinician participants watched a 10-minute recorded presentation on the evidence for each element, the guideline sources, and the rationale for using the element in the study. Additionally, clinicians were given a pre- and post-survey to measure their knowledge, attitudes, beliefs, and practices related to each of the seven elements.

### Quantitative data

#### Element observation

Seven SSI-prevention elements were monitored over a 12-month period (October 1, 2020 through September 31, 2021). Site coordinators collected data on the use of each element in eligible surgeries and entered the data into electronic case report forms.

Eligible surgeries included adult, non-emergency, non-trauma, colon surgeries. Sites were also provided with a list of International Classification of Diseases (ICD)-10 codes that identified eligible surgeries (available upon request).

Research Electronic Data Capture (REDCap), a third-party vendor that provides a password-protected, Health Insurance Portability and Accountability Act (HIPAA)-compliant electronic data capture platform, was used for remote data monitoring. Site coordinators were trained on using REDCap and the data collection elements. During data collection, validation procedures were used in the REDCap system and through SAS (SAS Institute, Cary, NC) to identify data entry errors, such as duplicate entries.

Study coordinators identified eligible cases by querying the electronic medical record (EMR) and daily surgery lists. The elements used were searched in the EMR and included anesthesia records, physician order entries, and other relevant records. They also conducted surgeon interviews if a particular element was not documented. Using REDCap, sites documented the use of one or more of the seven study elements. In addition to the data elements, site coordinators entered information related to age and gender, indications for surgery, type of surgery (i.e., open, laparoscopic, robotic), description of surgery, and lead surgeon.

### Qualitative data

#### Peer-to-peer calls

Five peer-to-peer calls were conducted with study coordinators and principal investigators (PIs). Each site presented a standardized slide deck to their peers that included a group discussion on their obstacles to implementing and documenting the seven elements, as well as recent successes to address them. These sessions generated discussion captured in transcripts and were analyzed for dominant themes.

#### Clinician surveys

**FIG. 1.**
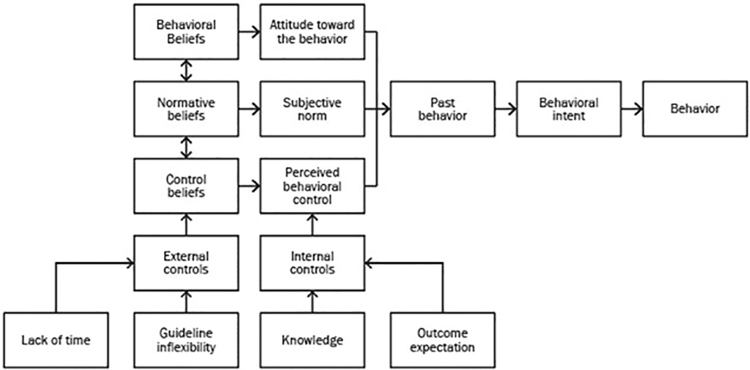
Behavioral change model developed based on three theoretical frameworks.

The pre- and post-study surveys were designed using constructs from Ajzen's Theory of Planned Behavior.^18^ This behavioral theory is used to predict and explain behaviors and has been used successfully in other studies to understand why evidence-based practices are not adopted into clinical care. A Physician Guideline Compliance Model^14^ was also used to add past behavior as an additional construct. It incorporates the physician's internal (knowledge, outcome expectation) and external (lack of time, guideline inflexibility) obstacles to guideline implementation. Another model construct incorporated was from the Development of an Evidenced Based Practice Questionnaire for Nurses.^19^ Constructs from these three models were synthesized to develop a unique behavioral change model, as shown in Figure 1.

A seven-point Likert scale collected and quantified results on clinicians' beliefs, perceived control, and intention to continue using the SSI-prevention elements. Survey questions and the constructs they represent are shown in Table 2.

**Table 2.** Clinician Survey Questions and the Constructs They Represent

Clinician survey questions; repeated for each of seven study elements	Theory construct
I believe this element would reduce SSI rates in my patients undergoing colon surgeries.	Behavioral belief
Most of my colleagues use this element in colon surgeries.	Normative belief
I believe it would be easy for me to implement this element for colon surgeries.	Control beliefs, internal
It is up to me if this element is used before colon surgeries.	Control beliefs, external
In the past, I have routinely used this element in colon surgeries.	Past behavior
In the future, I intend to use this element in colon surgeries.	Behavioral intention
This element has been proven to reduce SSI in colon surgeries.	Knowledge, outcome expectation

SSI = surgical site infection.

#### Study coordinator surveys

Upon completion of the study, study coordinators were asked to identify the top three obstacles and drivers for each element. These anonymous surveys were administered electronically through Premier's QUAD application. All responses were recorded as open text fields, and results were analyzed for dominant themes.

## Results

### Quantitative data: Element observation

Final individual and aggregate data were analyzed (Table 3). Lack of documentation was the most frequent response given for non-compliance of a particular element. When this was selected, the results were unable to distinguish whether an element was used.

**Table 3.** Study Element Documentation

	Element met							
	Total number of observed cases	Skin preparation *n *(%)	IV antibiotic agents *n *(%)	Triclosan sutures *n *(%)	Blood glucose *n *(%)	Body temperature *n *(%)	Order pre-operative MBP + oral ATBs *n *(%)	Oxygenation* n *(%)
Site one	319	319 (100)	305 (95.6)	176 (55.2)	263 (82.4)	40 (12.5)	122 (38.2)	3 (0.9)
Site two	277	276 (99.6)	277 (100)	277 (100)	243 (87.7)	171 (61.7)	18 (6.5)	61 (22.0)
Site three	262	261 (99.6)	253 (96.6)	212 (80.9)	49 (18.7)	30 (12.9)	18 (6.9)	16 (6.1)
Aggregate sum	858	856 (99.8)	835 (97.3)	650 (75.8)	555 (64.9)	443 (51.6)	158 (18.4)	80 (9.3)

IV = intravenous; MBP = mechanical bowel preparation; ATB = antibiotic agent.

The application of the skin prep and intravenous antibiotic agents were documented for nearly 100% of cases. Triclosan sutures were documented at 61% utilization when all sites were combined. However, site two reported 100% compliance, which they attributed to a unit policy to only stock triclosan sutures. The oxygenation element had a 9% compliance rate. In most cases, oxygenation was detailed. However, concentration or duration were not met because of an institutional policy to wean the patient to room air.

Blood glucose measure and body temperature elements were often not met because of the measure falling out of bounds. Overall, 65% had documentation that blood glucose was controlled, and 52% had documentation that normothermia was maintained.

The three institutions had significant difficulty locating documentation for a pre-operative mechanical bowel preparation and oral antibiotic order. The low documentation resulted in an 18% compliance rate. Because of high compliance rates with skin antiseptic preparations and intravenous surgical prophylaxis, further discussion is not warranted.

#### Triclosan sutures

Site two recorded 100% compliance because of standardization to triclosan-coated sutures in the peri-operative setting. Site one had detailed documentation of suture use however, they had the lowest compliance rate.

#### Blood glucose

Site three's unit policy for monitoring blood glucose was only for cardiac patients. They had the lowest compliance rate (19%).

#### Body temperature

Both site one and site three failed to meet compliance rates mostly as a result of failure to keep body temperatures within prescribed element range.

#### Mechanical bowel preparation plus oral antibiotic agents ordered

Sites two and three had the lowest compliance with documented administration of mechanical bowel preparation and oral antibiotic usage. However, overall compliance was unexpectedly low at 18%.

### Qualitative data

Analysis of transcript data from peer-to-peer and site management calls focusing on data extraction revealed documentation challenges at all sites as described below.

### Oxygenation

The PI at site one questioned the evidence for this element based on recent literature and push-back received from anesthesiologists and colorectal surgeons. This feedback included the lack of oxygenation as a current performance measure: *“*When it was a Surgical Care Improvement Project (SCIP) measure, they paid attention to it. It's not a SCIP measure, and so they don't pay attention to it.”

### Mechanical bowel preparation and oral antibiotic orders

All three sites reported difficulty with documentation of the pre-operation order for mechanical bowel preparation and oral antibiotic. Study coordinators searched the EMR, including the operative notes, medication orders, pre-operative paperwork, nursing notes, and progress notes, and sites also queried key search terms such as “GoLYTELY^®^.” The PI at site two remarked, “Some surgeons say we don't need to do this anymore, while others do it all the time,” and questioned where the evidence was for mechanical bowel preparation and oral antibiotic agents.

### Body temperature above 36.5°C

The normothermia element raised questions about standardizing the measurement of body temperature. Sites reported using a variety of measurement devices, including a forehead strip and an axillary thermometer. Two sites noted that normothermia was dropped as a SCIP performance measure and therefore was not documented as it once was. The coordinator at site three noted that their policy was to maintain body temperature above 36°C (versus the study's 36.5°C). The PI at site one said that although this was a “vital element,” they thought that less than 50% of surgeries at their hospital met this measure.

### Triclosan sutures

The PI at site one remarked, “Surgeons don't know what sutures they are using. Therefore, the best way to ensure they use triclosan sutures is to have a policy to only stock these sutures.” Another stated, “There's not a very good way for our nurses in the room to document supply usage. We are not currently scanning everything, so it's kind of document by exception.” Of note, site two had 100% compliance with the use of the sutures because of standardization at their institution.

### Blood glucose

Although all sites had lower-than-expected compliance rates with blood glucose monitoring, site three noted a conflict between their hospital policy and guideline recommendations because they only monitored blood glucose for cardiac patients based on prior SCIP measures.

### Local policies

Although all sites agreed with guideline recommendations as study elements, PIs noted that some measures conflicted with their existing local policy, such as oxygenation, blood glucose, and body temperature.

### Standardization of practices

When PIs were queried on how to best improve practices, one noted a unit policy to only stock triclosan sutures, however, they found these sutures were not purchased.

#### Clinician surveys

Twenty-eight of 29 consented clinicians completed the initial pre-survey, and 10 completed the post-survey. The low post-survey return rate was likely influenced by staffing issues during the pandemic. However, even with a lower post-survey rate, the groups were comparable. In the pre- and post-survey responses shown in Table 4, the most significant change in behavioral belief and outcome expectations was found for triclosan sutures (4.14–5.1) respectively. The oxygenation element scored second lowest for behavioral belief, outcome expectations, knowledge, and behavioral intent. Respondents consistently scored external control lower than internal control, even for elements with high behavioral and outcome beliefs and behavioral intention, such as skin preparation.

**Table 4.** Pre- and Post-Study Clinician Survey Likert Responses Averaged; Seven-Point Scale

	Behavioral belief, outcome expectation		Normative belief		Control beliefs, internal		Control beliefs, external		Past behavior		Behavioral intent		Knowledge, outcome expectation	
Model construct	Pre-study	Post-study	Pre-study	Post-study	Pre-study	Post-study	Pre-study	Post-study	Pre-study	Post-study	Pre-study	Post-study	Pre-study	Post-study
IV antibiotic agents	6.6	7.0	6.4	6.2	6.3	6.2	4.9	4.1	5.5	6.4	5.9	5.5	6.1	6.0
Triclosan sutures	4.1	5.1	4.1	4.6	4.7	4.8	4.4	4.1	3.4	4.0	4.4	4.5	4.5	4.3
Blood glucose	6.5	6.7	5.7	5.6	5.7	5.2	4.8	4.5	5.3	4.7	5.6	5.2	6.0	5.2
Body temperature	6.0	6.9	5.7	6.1	5.6	6.1	4.4	4.6	5.3	4.9	5.6	5.3	5.9	5.6
Oxygenation	4.7	5.2	4.3	4.4	4.9	4.8	4.5	4.1	3.9	4.0	4.6	4.4	4.7	4.8
Skin preparation	6.5	6.9	6.5	6.9	6.4	7.0	5.4	6.0	6.5	6.9	6.6	6.8	6.0	6.7
MBP + oral ATBs	6.1	5.9	5.5	5.7	5.6	5.6	5.3	5.3	5.3	5.3	5.7	5.8	5.9	5.7

IV = intravenous; MBP = mechanical bowel preparation; ATB = antibiotic agent.

In the post-survey, clinicians were asked, “What are the top three strategies that would enable you to use this study element more often?” For six of the seven study elements, a new unit policy was among the top responses. The only element for which this was not a top response was skin preparation. In addition, the only element for which surgeons needed more evidence that this strategy reduced SSIs was the triclosan sutures, which have extensive evidence of support for SSI prevention.^21–23^

#### Study coordinator surveys

We received two responses from study coordinators at each site. They identified the top drivers and obstacles for using each of the elements, shown in Table 5.

**Table 5.** Study Coordinators' Post-Survey Responses

Element	Drivers for Adoption	Obstacles for Adoption
IV antibiotics	Established standard of care	None identified
Triclosan sutures	Required by many policies so it is standard practice Independently validated Easy to implement Triclosan-coated sutures are available in the operating room. Being the only thing available. I do not think any of the surgeons actively thought about using them. Easy to get a hold of	They do not come in the non-absorbable kind of suture Surgeons probably do not think about them, they just use what is available Staff probably do not know what they are Many sutures are available in the operating room and many different kinds of sutures are used for each procedure It seems that surgeons may not be sure if the suture is coated with triclosan.
Blood glucose	Each patient is monitored very closely.	Lack of knowledge Not part of many SSI bundle policies Known patients with diabetes mellitus are really the only ones I saw being checked
Body temperature	People know the patient is supposed to be kept warm Warm blankets Forced air blankets	Surgery suites are usually very cold Sometimes the temperature may just be 0.1–0.5°C 36.5°C.
Oxygenation	Nasal cannula is readily available.	Staff are taught to wean patients off O_2_ ASAP
Skin preparation	Established standard of care	None identified
Order MBP + oral ATBs	Established standard of care	Inadequate documentation Surgeons not ordering this, just clear liquid diet and IV antibiotic agents Patients not being compliant

IV = intravenous; MBP = mechanical bowel preparation; ATB = antibiotic agent; SSI = surgical site infection.

## Discussion

Evidence-based SSI prevention strategies have been updated in guidelines since 2016 (WHO,^4^ ACS^20^) and 2017 (CDC^6^). Until now, there has been little evidence of clinical implementation of these guidelines or the obstacles and drivers to adopting them. This observational study set in three large, geographically diverse hospital systems demonstrated the challenges that institutions and staff encounter when implementing guidelines into practice.

### Standardization of practices

Behavioral change is often difficult to implement in any environment, particularly in the peri-operative setting. This often leads to inconsistent implementation and a potential lack of standardization because it can take significant effort and time to change clinical practice. One option offered is to focus on standardization by eliminating variability (i.e., choice) to move a particular clinical practice closer to guideline recommendations.

In this observational study, strategies to improve standardization began with policy development. Policies were a pivotal aspect of site discussions on how to implement the SSI prevention elements. This was the top recommendation from surgeons to change a practice or choice. For example, the PI at site 2 mentioned that introducing a standing order for mechanical bowel preparation plus oral antibiotic agents when the patient's surgery is scheduled would improve compliance. When eliminating choices, it is beneficial to the implementation of standardized practices in the peri-operative environment.

### Behavioral change model

Among all study elements, glycemic control, intravenous antibiotic agents, and triclosan sutures have the strongest evidence-based research. However, the colon surgeon's surveys demonstrated they had the least belief in the triclosan sutures, which have been shown in multiple systematic reviews and meta-analyses to result in a 20% to 56% decreased risk of SSIs.^21–23^

Examining how belief in an evidence-based practice could be improved, Dobler et al.^24^ suggest that belief should be framed as a cognitive bias. These authors discuss “real-time workplace strategies” to make changes at a system level versus an individual surgeon's level to eliminate cognitive bias. These changes may be as simple as a surgical checklist or more structured with product standardization and reducing choices.

The study model and clinician surveys reported that implementing SSI prevention elements was easy to do (i.e., high internal control construct). However, implementation was not in their jurisdiction (i.e., low external control). Additionally, surgeons acknowledged that unit policies (external) were the best way to implement the guideline elements.

In institutions where study elements consistently demonstrated high compliance, such as skin preparation, it highlighted their leaders' acceptance of the responsibility for standardization and guideline compliance. Ideally, future research could examine how to adopt other guideline elements and make them as fundamental as skin preparation.

### Documenting the study elements and measurement techniques

The fundamental principle of quality improvement is documentation in the EMR. Documentation for many of the elements in the EMR was found to be missing, resulting in the inability to validate if an element was used. Whenever a new policy, practice, or procedure is implemented, a key step should be to identify and educate where and how to document the element in the EMR. When a key element is not ordered or intentionally omitted, the reason should be documented. This permits quality improvement to measure and evaluate results.

Although documentation was lacking, standardizing measurement techniques was another important discussion point to compare processes and outcomes. Sites discussed how certain elements were measured and the potential unreliability with measurement comparisons. For example, one site used a forehead strip to measure body temperature while another used the axillary temperature, and the third was unsure how body temperature was measured.

### Strengths of the study

Triangulated qualitative data was used in this study design. Specifically, we analyzed peer-to-peer call transcripts, site management calls, clinician surveys, and site coordinator surveys to identify and confirm dominant themes in the data. The clinician survey was developed using a well-published theory, and we operationalized those constructs using published survey studies on changing clinician practices. Finally, even with the coronavirus disease 2019 (COVID-19) pandemic potentially negatively affecting the number of eligible colon surgeries, each site completed its data entry on more than 225 eligible cases, meeting and exceeding the pre-COVID-19 expectations.

### Limitations of the study

The study was unable to distinguish whether a guideline element was used during surgery due to lack of documentation. For example, lack of documentation was noted in all observed study elements except for skin preparation and intravenous antibiotic agents. This was a major finding and merits further exploration when trying to change clinical practices.

The three sites used different techniques and definitions when documenting body temperature or suture use at different layers. Because of the lack of a standardized measurement technique and local interpretation of observed elements, this study is unable to demonstrate that the results were comparable. It does, however, identify the need for standardized measurement techniques and documentation in the EMR when implementing guideline elements in the peri-operative setting and other patient care areas.

The site coordinators recognized that there were some situations in which clinicians and staff were following a standard of care policy that did not require documenting a certain element. When these situations exist in an institution that is working to improve SSI prevention strategies, leadership needs to recognize and address how improvements will be documented. It must be noted that this study was observational, and we did not ask sites to document element use for a quality improvement initiative. Instead, our results reflect current real-world practices.

## Conclusions

Six years after strong evidence-based guidelines were published for SSI prevention, there is scant evidence they are being implemented. This observational study underscores the difficulties in implementing SSI evidence-based guidelines in perioperative settings. The study revealed a lack of accurate and consistent documentation across all three sites. This is a major finding that needs urgent attention in to effectively implement evidence-based guidelines focused on reducing SSIs.

Enforcing institutional policies was identified as the primary way to change practice and implement guidelines. However, while certain policies such as standardizing triclosan sutures may result in an easy change in clinician behavior, others may take more work, such as oral antibiotics and mechanical bowel preparation.

Further research is needed using a structured approach to develop strategies for the implementation of evidence-based guidelines. This will enable healthcare organizations to provide quality care to surgical patients by mitigating sentinel patient risk factors. We have compiled a list of implementation strategies (Supplementary Table S1). This shows possible implementation strategies of the seven guideline elements that were used in this study and their references.

Although this observational study was initiated before the pandemic, it clearly identified issues and challenges healthcare organizations often encounter when they fail to fully adopt standardized guidelines. Every surgical patient should receive the best, evidence-based interventions on every occasion, at the right time, and therefore, healthcare institutions must document that this has been done in a timely fashion.^33^

## Supplementary Material

Supplemental data
