# Supplementary material for: Effectiveness versus Uptake: The Challenges of Implementing Evidence-Based Strategies to Reduce Surgical Site Infection in Patients with Colon Surgeries
Source: Surg Infect (Larchmt). 2023 Apr 27;24(4):382–9. doi: 10.1089/sur.2022.411 (PMC10173747; doi:10.1089/sur.2022.411)
Supplement: Supplemental data [file Suppl_TableS1.docx]

Supplementary Table S1. Team Recommendations on Successful Implementation of the Seven Elements

| **Element** | **Guideline Recommendations** | **Suggested Implementation Strategies** |
| --- | --- | --- |
| IV Antibiotics *^a,c,d,e^* | Policy/protocol includes documentation of:   \| - Administer preoperative antimicrobial agents only when indicated, based on published clinical practice guidelines - In clean and clean-contaminated procedures, do not administer additional prophylactic antimicrobial agent doses after the surgical incision is closed in the operating room, even in the presence of a drain \| \| --- \| | - Develop standardized order sets within the Electronic Medical Record (EMR) - Conduct education campaign with surgeons, PA, and NP’s - Administer and document in the EMR that antimicrobials are administered per policy - Use performance improvement tools to review the process pathway and revise if necessary |
| Triclosan coated sutures *^a,c,d,e^* | Policy/protocol includes documentation of:   - The routine use of antimicrobial coated sutures at the deep layer, organ layer and superficial layer | - Standardize to antimicrobial coated sutures within surgical services - Update surgeon preference cards to reflect the current triclosan coated suture policy - Validate antimicrobial coated sutures are stocked and available within the operating room (cart or on shelves) - Request in-service from company reps (Ethicon) - Conduct an observational study of a cohort of colorectal procedures to determine adoption of standards - Conduct focused education for outliers |
| Glycemic control *^a,c,d,e^* | Policy/protocol should include documentation of:   - Implement perioperative glycemic control and blood glucose target levels of <200 mg/dl in diabetic and non-diabetic surgical patients. | - Include documentation of perioperative glycemic control into the Electronic Medical Record (EMR) - Conduct education campaign with surgeons, PA, NP’s, and staff - Conduct a quality assessment of a cohort of surgical procedures to determine current compliance with practice issues - Conduct focused education for outliers |
| Normothermia *^a-e^* | Policy/protocol includes documentation of:   - Body temperature measurement should be standardized - Maintain perioperative normothermia at >36oC or 36.5 | - Standardize use of temperature measurement devices - Use facility approved warming devices preoperatively, intraoperatively, and in the post anesthesia care unit - Use warmed fluids intra-operatively - Apply hats and booties preoperatively - Conduct education campaign on normothermia with preop staff, anesthesia, and PACU - Include documentation of body temperature into the Electronic Medical Record (EMR) - Monitor engineering controls to maintain the operating room temperature within recommended parameters |
| Oxygenation ^a,b,c,d,e^ | Policy/protocol defines adequate postoperative oxygenation as:   \| - Oxygen supplementation (80% FiO2) during the perioperative period has been documented to reduce the risk of SSI in patients undergoing colorectal surgeries is controversial.(27, 28) - Documentation of administration supplemental oxygen (80% FiO2) after surgery performed under general anesthesia. (ACS) \| \| --- \| | - Assess current institutional policies on supplemental oxygenation after general anesthesia and revise if necessary - Conduct a quality assessment of a cohort of surgical procedures to determine current compliance - Conduct staff training (If compliance is low) with anesthesia, PACU, and receiving nursing units. - Include documentation of supplemental oxygenation into the Electronic Medical Record (EMR) |
| Skin prep *^a,b,c, d, e^* | Policy/protocol includes:   - Standardization to alcohol-based skin prep solution, and - A standardized application procedure for the alcohol-based skin prep. - If alcohol cannot be included in the skin preparation, then use chlorhexidine gluconate instead of povidone iodine, unless contra-indications exist. (ACS) | - Documentation of the selected skin prep in the electronic medical record (EMR). - Conduct an observational study of a cohort of surgical procedures to determine standardized application technique. - Monitor staff performance periodically and conduct additional training as necessary - Periodic in-services from company reps on proper antiseptic application. - Validation of staff’s performance should be an element of new hire orientation. - Review and update surgeon’s preference card to align with institution policy |
| Order MBP + oral ATBs *^a,d,e,f^* | Policy/protocol includes:   - Preoperative oral antibiotics in combination with mechanical bowel preparations (OA-MBP) as a safe and effective adjunctive strategy for reducing the risk of infection following colorectal surgery.(29-33) - Current peer-reviewed evidence indicates that OA-MBP should be part of a comprehensive colorectal surgical care bundle.(29-34) | - Develop standardized order sets within the EMR - Review the process for patient education to perform the bowel prep prior to surgery - Conduct an observational study of a cohort of surgical procedures to determine the preoperative oral antibiotics in combination with mechanical bowel preparations. - Conduct education campaign with surgeons, PA, NP’s, and nursing - Document in the EMR that MBP + oral ATBs be given/taken prior to surgery |

1. American College of Surgeons (ACS) and SIS; SSI guidelines 2016
2. Association of PeriOperative Registered Nurses (AORN) Guidelines for Perioperative Practice – Hypothermia 2019
3. CDC SSI guidelines 2017-2018
4. Wisconsin SSI Guidelines 2017
5. APIC Implementation Guide to the OR
6. Migaly J, Bafford AC, Francone TD, Gaertner WB, Eskicioglu C, Bordeianou L, Feingold DL, Steele SR; Clinical Practice Guidelines Committee of the American Society of Colon and Rectal Surgeons. The American Society of Colon and Rectal Surgeons Clinical Practice Guidelines for the Use of Bowel Preparation in Elective Colon and Rectal Surgery. Dis Colon Rectum. 2019 Jan;62(1):3-8. doi: 10.1097/DCR.0000000000001238. Erratum in: Dis Colon Rectum. 2019 Oct;62(10):e436. PMID: 30531263.
